# Real-World Open-Label Experience with Rimegepant for the Acute Treatment of Migraine Attacks: A Multicenter Pilot Study

**DOI:** 10.3390/brainsci14121169

**Published:** 2024-11-22

**Authors:** Emmanouil V. Dermitzakis, Dimitrios Rikos, Michail Vikelis, Georgia Xiromerisiou, Styliani Zisopoulou, Dimitrios Rallis, Panagiotis Soldatos, George S. Vlachos, Georgios G. Vasiliadis, Andreas A. Argyriou

**Affiliations:** 1 Euromedica General Clinic, 54645 Thessaloniki, Greece; georgios.vasiliadis@yahoo.com; 2 404 Military Hospital, 41222 Larisa, Greece; rikosd@hotmail.com; 3 Glyfada Headache Center, 16675 Glyfada, Greece; 4 Department of Neurology, School of Medicine, University of Thessaly, 41110 Larissa, Greece; georgiaxiromerisiou@gmail.com; 5 Clinic Calypso, 41222 Larisa, Greece; neurozisopoulou@gmail.com; 6 Neurology Clinic, Peripheral General Hospital Tzaneio, 18536 Peiraias, Greece; jimrallis@hotmail.com; 7 Independent Researcher, 24100 Kalamata, Greece; soldatosp@gmail.com; 8 Private Practice, 15231 Chalandri, Greece; gvlachos@neuromed.gr; 9 Headache Outpatient Clinic, Neurology Department, Patras Agios Andreas General Hospital of Patras, 26335 Patras, Greece; andargyriou@yahoo.gr

**Keywords:** migraine, acute treatment, gepants, rimegepant

## Abstract

**Objectives:** The present open-label multicenter pilot study sought to prospectively evaluate the efficacy and safety of rimegepant in treating migraine attacks. **Methods:** The primary endpoint was pain freedom at two hours post-dose, while the co-primary efficacy endpoints included a reduction in the headache intensity and freedom from the most bothersome symptoms (MBS) associated with migraine at the same time point. To test the potential efficacy of rimegepant, patients were asked to record in a questionnaire all the relevant changes with each migraine attack treated with rimegepant at two hours post-dose vs. two hours before. The attending neurologists provided information on the basic demographics, medical anamnesis, and migraine history as well as the triptan use history. **Results:** A total of 54 patients (32 with episodic and 22 with chronic migraine) received rimegepant 75 mg at least once during a single migraine attack (overall, 140 dosage intakes). Pain freedom at 2 h was achieved in 45/140 (32.1%) intakes. Regarding the efficacy of the first rimegepant dose (n = 54), significant reductions in the headache intensity were observed between the pre- and 2 h post-treatment average VAS scores (−4.8 ± 2.8 mean; *p* < 0.001). Likewise, the same mean reductions in the average VAS scores occurred when the 2 h response to all 140 doses was analyzed (−5 ± 2.8; *p* < 0.001). Freedom from MBS at 2 h post-dose was achieved for photophobia in 43%, for phonophobia in 53%, and for nausea in 57%. The ability to fully return to everyday activities at 2 h post-dose was achieved in 83/140 instances (59%). We only recorded mild adverse events in 24/140 dosages. **Conclusions:** Our preliminary results demonstrate that rimegepant is effective, safe, and well tolerated in treating acute migraine attacks.

## 1. Introduction

According to the most recent iteration of the International Classification of Headache Disorders, Third Edition (ICHD-3), migraine is defined as a recurrent headache that is characterized by at least two attacks with transient focal aura or five attacks without aura of usually unilateral moderate/severe throbbing pain or a pulsating sensation lasting between 4 and 72 h [1]. In addition, it should be aggravated by routine physical activity and it should be accompanied by at least one associated symptom, including nausea, vomiting, and extreme sensitivity to light or noise [1].

Considering its monthly frequency, migraine can be classified as episodic migraine (EM) if <15 headache days per month (MHDs) occur or chronic migraine (CM), where headache days are ≥15/months, of which at least 8 can be characterized as migraines or respond to migraine-specific medications, either triptans or gepants, for at least 3 consecutive months [1,2]. Furthermore, EM is further subdivided into low-frequency if 4–7 MHDs occur and high-frequency migraine if 8–14 MHDs occur [2].

Almost all patients are in need of symptomatic treatment during acute migraine attacks. However, clinical practice shows that there are significant unmet needs with the use of various triptan formulations, ergots, simple analgesics, nonsteroidal anti-inflammatory medications (NSAIDs), and paracetamol/acetylsalicylic acid/caffeine combinations for symptomatic migraine management [3,4], because of the low percentages of pain freedom at 2 h post-dose, inadequate headache intensity reduction, and/or poor tolerability to these acute treatment options [5]. Freedom from the most bothersome symptoms (MBSs), such as nausea/vomiting and photo-/phonophobia [6] at two hours post-dose also ranks among the open challenges in effectively treating acute migraine attacks [7].

Nonetheless, triptans represent the standard of care in the treatment of moderate/severe acute migraine attacks and associated symptoms through the selective affinity for 5-hydroxytryptamine (5-HT)_1B_ and 5-HT_1D_ serotonin receptors and the variable activity on 5-HT_1F_ receptors. Moreover, the mode of triptans’ action is based on the ability to prevent neurogenic vasodilation and inflammation and to inhibit the central sensitization of trigeminal neurons through vasoconstrictive effects and the inhibition of the release of several neuropeptides and neurotransmitters, such as glutamate, neurokinin A, calcitonin gene-related peptide (CGRP) and substance P [8]. Oral formulations of triptans, including sumatriptan, almotriptan, eletriptan, frovatriptan, naratriptan, rizatriptan, and zolmitriptan, show strong evidence for efficacy, although eletriptan and rizatriptan seem superior because of their highest 2 h and 24 h pain-free rates, compared to other triptans [9]. However, their use is hampered by common evidence of medication overuse headache (MOH) due to tachyphylaxis, which means a progressive decrease in response after repeated administration of treatment in some patients [10]. In addition, many patients poorly tolerate triptans due to evidence of adverse events, including nausea; dry mouth; dizziness; flushing; hand and feet paresthesia; face, limb, and chest pressure; and somnolence. Finally, triptans are contraindicated in patients with major or uncontrolled cardiovascular complications [11].

Nonetheless, the competitive landscape of acute migraine treatments has continuously evolved over the past few years. As such, new acute pharmacological strategies for migraine are available, including gepants, which are small molecules targeting the CGRP receptor. Gepants do not cause vasoconstriction and have generally less tolerability issues, appearing as such superior in terms of safety over triptans, particularly in patients with an increased risk of cardiovascular comorbidities. In addition, gepants do not seem to cause medication overuse headache [12].

Rimegepant, an orally administered gepant currently approved in 68 countries, was approved in the United States in 2020 as the only symptomatic and preventative treatment for migraine in adults, whereas it received a marketing authorization valid throughout the European Union on April 2022 [13]. Rimegepant binds to the CGRP receptor, thus preventing CGRP from being attached to the receptor. The recommended dose is 75 mg as needed up to once a day for acute migraine attacks in adults and 75 mg every other day for the preventive treatment of episodic migraine in adults [13]. Rimegepant 75 mg is absorbed with a time to maximum concentration of 1.5 h and a half-life elimination at 11 h. Moreover, rimegepant is primarily metabolized by CYP3A4 and to a lesser extent by CYP2C9 and, as such, it is advised to avoid its co-administration with strong inhibitors of CYP3A4 and CYP2C9 as well as strong or moderate inducers of CYP3A [13].

Rimegepant was extensively studied in the context of different randomized controlled trials (RCTs) and was found to achieve a higher percentage of patients with pain freedom who were also free from MBSs than that with placebo at 2 h post-dose [14]. A study in 38 healthy volunteers also showed that a single dose of rimegepant (up to 300 mg) does not have a clinical effect on electrocardiogram parameters [15].

In line with the Greek reimbursement policies currently applied for high-cost therapies, gepants, including rimegepant, are not reimbursed by the Greek National Health System for either the acute or prophylactic treatment of migraine and patients have to pay out of pocket in order to maintain therapy with rimegepant. While awaiting full reimbursement of rimegepant for acute migraine treatment, we report the outcomes of a pilot real-world open-label study that evaluated the efficacy of rimegepant for treating acute migraine.

## 2. Materials and Methods

In this multicenter, prospective, open-label pilot study, we enrolled patients who were followed up with on their migraines in seven headache centers located in five different major urban Greek geographical areas, including Attica (Athens), Central Macedonia (Thessaloniki), Thessaly (Larissa), and Peloponnese (Patras and Kalamata). All participants signed an informed consent form prior to their enrollment and the study protocol was approved by the Institutional Review Board of “Euromedica” General Clinic, Thessaloniki, Greece (750/05-05-2023), in accordance with the Declaration of Helsinki.

Eligibility was confirmed by a protocol-specific checklist. To be eligible for enrollment, patients had to be diagnosed with either episodic (EM) or chronic (CM) migraine, according to the most updated version of the diagnostic criteria of the International Classification of Headache Disorders [1]. It was also mandatory to not fulfill any exclusion criteria or have any contraindication to rimegepant (brainstem aura, active hepatic disease, intake of strong cytochrome 3A4 inhibitors/inducers, etc.), as outlined in the approved summary of the product’s characteristics [13]. Those taking rimegepant (Vydura^®^ oral/lyop 75 mg/tb, Pfizer-Athens, Greece) at least once, self-administered as needed, for the acute symptomatic treatment of a migraine attack (with or without aura) of any severity (mild, moderate, or severe headache pain intensity) were eventually enrolled and prospectively evaluated. Being a pilot open-label study, no randomization or use of controls was applied.

To test the potential efficacy of rimegepant, patients were asked to record in a questionnaire all the relevant changes with each migraine attack treated with rimegepant at two hours post-dose vs. two hours before. The clinical report form used for the purposes of this study is available as Supplementary Material. The primary endpoint was pain freedom. Co-primary efficacy endpoints consisted of the reduction in headache intensity and freedom from the MBSs associated with migraine, including nausea/vomiting and photo-/phono-phobia; all of the above at two hours post-dose were compared to baseline (rimegepant intake). To test the potential efficacy of rimegepant, patients were asked to record in a questionnaire all relevant changes at each migraine attack treated with rimegepant at two hours post-dose vs. two hours before. Pain freedom and freedom from the MBSs were defined by calculating the proportion of patients reporting no pain or absence of MBSs at two hours post-dose. The analyzed MBSs included photophobia (sensitivity or aversion to light), phonophobia (sensitivity or aversion to sounds), and nausea (urge to vomit) [16]. The reduction in peak headache intensity was assessed using a visual analogue 0–10 numerical scale (VAS), before and at two hours, post-rimegepant. The ability to return to everyday activities at two hours post-dose was evaluated with a 3-point rating scale (0—no; 1—somewhat; 2—yes); the overall satisfaction with rimegepant was measured with a 4-point rating scale (0—not at all, 1—some, 2—much, 3—very much).

The attending neurologists also completed a questionnaire per patient regarding basic demographics, medical anamnesis, migraine history (low- or high-frequency EM and CM, and previous or current prophylactic anti-migraine treatments), as well as triptan use history. Those currently receiving prophylactic medication had to be on a stable dose for at least three months before inclusion.

Relevant comparisons were made between patients who reported failure of more than one triptan. Finally, the consistency of the treatment effect was also evaluated in the subgroup of patients that received at least two rimegepant doses for treating separate migraine attacks.

The assessment of rimegepant safety was based upon the number and percentage of participants with occurrence of adverse events (AEs) through 24 h post-dose. Patients were encouraged to report any AEs either spontaneously or in response to direct questioning through phone contacts. The Medical Dictionary for Regulatory Activities (MedDRA) version 21.1 was used. The relationship of each AE to rimegepant was determined by the treating physician and then classified as “related”, “possibly related”, “unlikely related”, and “unrelated”. AEs with study drug relationships of “related” and “possibly related” were counted as treatment-related AEs. The severity of AEs was rated as “mild”, “moderate”, and “severe enough leading to study drug discontinuation”.

The statistical significance was analyzed using a paired samples *t*-test in two groups of data, using SPSS for Windows (release 27.0; IBM, Chicago, IL, USA). The first group evaluated the efficacy of just the first dose (single rimegepant dose intake) that all the included patients received and the second included all the rimegepant doses received by each participant. Categorical variables, such as groups of patients, were analyzed with the chi-squared Fisher’s exact test. Unless otherwise stated, all tests were two-sided, and significance was set at *p* < 0.05.

## 3. Results

Overall, 54 patients (32 with EM and 22 with CM), with a mean age of 45.3 ± 12.8 years, received at least one dose of rimegepant 75 mg during a single migraine attack. Among them, 14 patients received one dose, 23 received two doses and 17 patients received more than two doses, resulting in a total of 140 documented doses for a corresponding number of distinct migraine attacks. From those 54 participants, 44 failed due to a lack of efficacy and/or poor tolerability to up to four different triptans. Among them, 16 (29.6%) had failed on one triptan, 16 (29.6%) on two triptans, 9 (16.7%) on three triptans and 3 (5.6%) on four triptans. Nine patients preferred to pay out-of-pocket and be treated with rimegepant, despite being triptan responders and having had no adverse events. The patients’ demographic characteristics are shown in Table 1.

Pain freedom at 2 h was achieved in 45/140 (32.1%) rimegepant intakes, while a significant percentage of patients achieved a 50% or 75% pain reduction post-dose. A less than 50% pain reduction (treatment failure) at 2 h post-dose was observed in just 26 of the total 140 dosages (19%). Freedom from the MBSs at 2 h post-dose was achieved for photophobia in 43%, for phonophobia in 53%, and for nausea in 57%.

**Figure 1:**
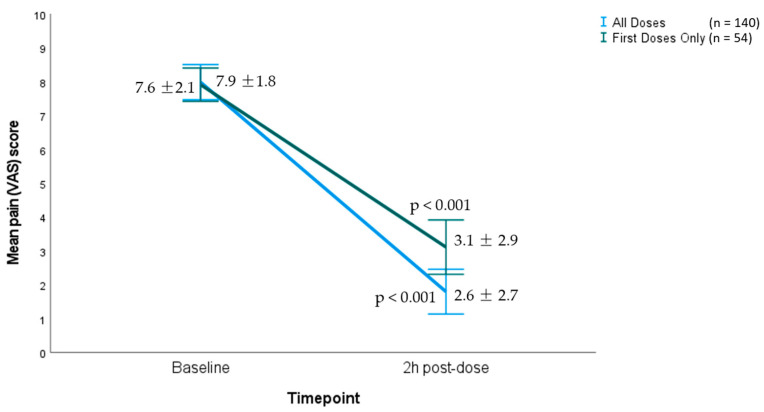
Changes in the intensity of pain, according to VAS, after the first rimegepant dose only (n = 54) and after all doses (n = 140).

Regarding the efficacy of the first rimegepant dose only in all the enrolled patients (n = 54), it was evident that the pre-treatment VAS score was 7.9 ± 1.8 and dropped to 3.1 ± 2.9 post-treatment; resulting in a −4.8 ± 2.8 mean reduction in the headache intensity (t: 12.75; *p* < 0.001). Likewise, rimegepant was deemed highly efficacious when the 2 h response to all 140 doses was analyzed and it was shown that the mean pre-treatment headache intensity VAS score was significantly reduced from 7.6 ± 2.1 to 2.6 ± 2.7 post-treatment (mean reduction: −5 ± 2.8; paired samples *t*-test: 21.25; *p* < 0.001). Figure 1 depicts the changes in the mean pain VAS scores between pre- and post-rimegepant intake.

**Figure 2:**
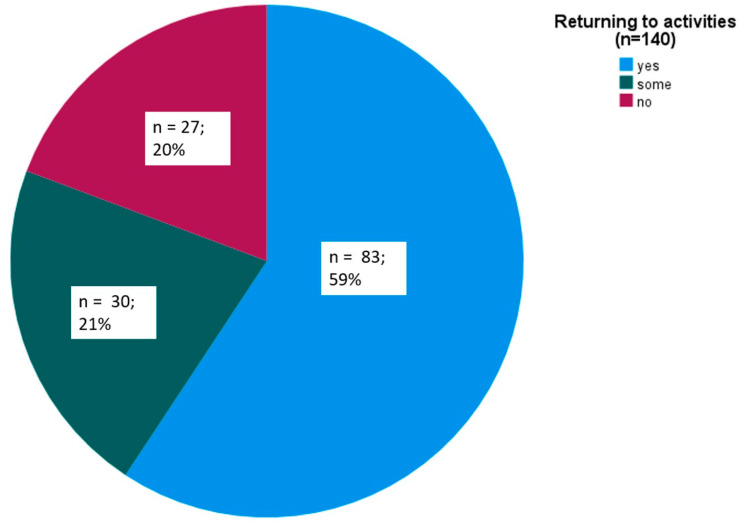
Ability of returning to everyday activities 2 h after all 140 rimegepant intakes.

Returning to everyday activities 2 h after all 140 rimegepant intakes for separate migraine attacks was achieved in 83 (59%) instances (Figure 2).

**Figure 3:**
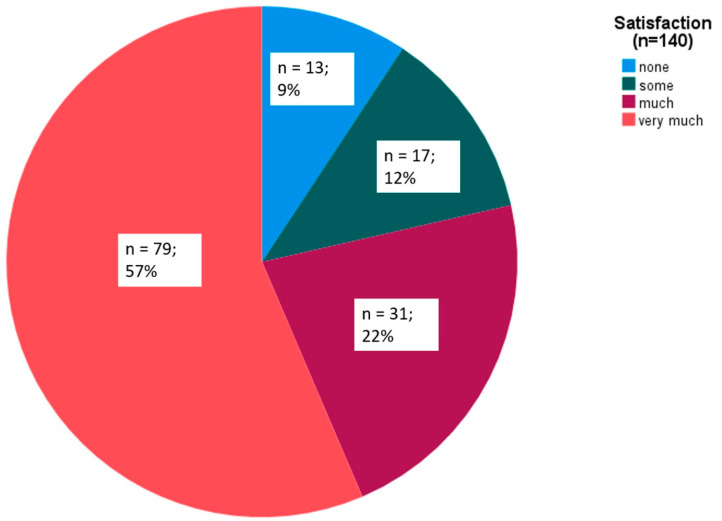
Satisfaction rates for all 140 rimegepant dosages.

The overall satisfaction of the patients with all the rimegepant intakes remained high, as the patients remained “very much” satisfied after 79 (57%) of all 140 rimegepant dosages (Figure 3).

The overall efficacy results, response rates, and patients’ satisfaction 2 h after a single (n = 54) or all (n = 140) rimegepant intakes are summarized in Table 2.

Concerning safety, no new rimegepant-related AEs were noted. Overall, we recorded mild rimegepant-related AEs in 24/140 dosages (17.1%), mainly including transient dizziness (n = 11), nausea (n = 7), and fatigue (n = 2). There were no cases of discontinuing treatment due to safety/tolerability issues, whereas none of the patients reported an intention to stop taking rimegepant in the future for such issues.

## 4. Discussion

CGRP-targeting acute therapies are generally expected to have better efficacy than that of conventional therapies because of their ability to diminish pain generation by inhibiting the vasoactive effect of CGRP release coupled with halting of the activation and sensitization of trigeminovascular nociceptors [17]. Rimegepant, belonging to the second-generation gepants, acts by selectively binding with high affinity to the human CGRP receptor to antagonize its function [18].

The results of the current pilot, open-label, multicenter study revealed that rimegepant 75 mg effectively provided clinically meaningful analgesia at 2 h post-dose in the majority of the total intakes, at 69/140 (49%), while pain freedom was observed in 32%. It was also effective in providing freedom from MBSs in a significant percentage of 140 single dosages.

The rates of both the ability to return to daily activities and the overall satisfaction was fairly high. Our results are in agreement with the findings from both RCTs and real-world evidence (RWE) studies, demonstrating that rimegepant has shown positive results over placebos in terms of the absence of migraine-associated symptoms at 2 h, sustained pain relief for 2–24 h, and tolerability, as well as safety in treating acute migraine attacks [19], even in patients with an insufficient response to ≥2 triptans [20]. Specifically, a dose-finding RCT showed that rimegepant was superior to a placebo at several different doses (75–300 mg) and had an excellent tolerability profile [21]. This study showed that rimegepant 150 mg was better than placebo for pain freedom at 2 h, while a higher total migraine freedom (2 h) was evident for rimegepant 75–300 mg and sumatriptan vs. placebo. There was no between-group difference in terms of the safety [21].

Another RCT reported that rimegepant 75 mg was superior to placebo at 2 h for all the primary outcomes, including pain freedom and the absence of migraine-associated symptoms (nausea, photophobia, and phonophobia) [22]. Moreover, a separate RCT, enrolling 1375 patients, of whom 1351 (mean age = 40.2 ± 12; F: 1147, M: 204) received treatment, showed that rimegepant 75 mg (n = 669) was superior to placebo (n = 682) for all the primary and secondary outcomes, except for freedom from nausea at 2 h and pain relapse [23]. Notably, in a recently published randomized placebo-controlled Phase 3 trial with 1458 participants [24], the percentage of rimegepant participants with pain freedom 2 h post-dose was 19.2%. In our study, the percentage of pain freedom 2 h post-dose was 32% for all intakes, which could suggest repeatable and consistent effectiveness.

Likewise, comparable results have been reported in all MBSs between our and the latter study [24]. Specifically, photophobia freedom at 2 h post-dose was seen in 34.9% of the rimegepant participants vs. 43% for all the intakes in our study (phonophobia freedom: 36.6% vs. 53%; nausea freedom: 46.9% vs. 57%). Moreover, several real-world evidence (RWE) studies confirm that rimegepant appears superior to placebo for pain freedom and absence of migraine-associated symptoms at 2 h, without between-group differences in terms of safety [25,26]. Nonetheless, head-to-head comparisons between rimegepant and triptans as well as other analgesics and NSAIDs are warranted to elucidate the cost–benefit ratio of this new class of abortive drugs.

The incidence and severity of the overall AEs noted in our patients were similar to those reported in previously published data, mainly for dizziness and nausea [24,26], whereas no new rimegepant-related safety signals were reported. Of note are the results of a study that investigated the real-world profile of gepant-associated AEs. This study analyzed data collected from the VigiAccess database and the U.S. Food and Drug Administration (FDA) Adverse Event Reporting System (FAERS) database. It was revealed that among all the gepants, rimegepant had the strongest signal for gastrointestinal AEs, skin and subcutaneous tissue disorders, alopecia, cardiac AEs, and Raynaud’s phenomenon [27]. In agreement with the above, and considering that safety issues are of paramount importance for therapeutic decision-making, future studies are needed to answer the clinically important question of whether rimegepant may have a better tolerability than triptans.

The small sample size studied and the open-label design are acknowledged as significant caveats of our pilot study, limiting the generalizability of our results both in terms of efficacy and safety. The national policy that is currently applied in Greece for reimbursing high-cost anti-migraine therapies restricted us from having a larger sample size, as many patients were reluctant to directly pay out of pocket a significant amount of money (EUR 66 for two tablets) for regularly maintaining rimegepant therapy for acute migraine. Indeed, patients’ care and treatment access can be affected by the choice of national reimbursement policies [28]. Particularly for gepants, its use can be considered pending various additional reimbursement restrictions as a third-line acute anti-migraine treatment when analgesics, NSAIDs and triptans are not effective, poorly tolerated, or contraindicated. As such, the delayed access to these medications may result in continued treatment with triptans or other unspecific medications; during which patients may remain unresponsive and also endure the chronification of their episodic migraine [29].

## 5. Conclusions

The results of our pilot study demonstrate that rimegepant is generally effective, safe, and well tolerated in treating acute migraine attacks. On clinical grounds, we could suggest that rimegepant seems able to provide clinically meaningful analgesia in migraine patients during attacks and also improve patients’ compliance with treatment. Considering that it is not cost-effective in all patients, further studies are needed to clarify if disparate plasma CGRP levels or differentiations in other factors might be able to predict the response to rimegepant in the symptomatic treatment of migraine.

## Figures and Tables

**Table 1:** Demographic characteristics of patients (n = 54).

Headache Type	
LFEM	23
HFEM	9
CM	22
Gender	
Female	45
Male	9
Age in years Mean ± standard deviation (min–max)	45.3 ± 12.8 (19–69)
BMI	
Av. (min–max)	24.5 (17.3–35.5)
Age at first migraine diagnosis	
Av. (min–max)	21.2 (7–41)
History of triptan use	
Yes	53
No	1
No. of failed triptans	
1 triptan, n (%)	16 (29.6)
2 triptans, n (%)	16 (26.6)
3 triptans, n (%)	9 (16.7)
4 triptans, n (%)	3 (5.6)

Table abbreviations: LFEM: low-frequency episodic migraine; HFEM: high-frequency episodic migraine; CM: chronic migraine; BMI: body mass index.

**Table 2:** Outcomes at 2 h after a single (n = 54) or all (n = 140) rimegepant intakes.

	Pre-Treatment	2 h Post-Treatment	
Change in pain VAS score (paired samples *t*-test)			Mean difference
Average in first dose (n = 54)	7.9 (1.8)	3.1 (2.9)	4.8 (*p* < 0.001)
Average in all doses (n = 140)	7.6 (2.1)	2.6 (2.7)	5 (*p* < 0.001)
			Patients who had the symptom and became symptom-free at 2 h post-dose
Photophobia (n = 122)			
No	18	70	52 (43%)
Some	26	54	
Much	32	7	
Very much	64	9	
Phonophobia (n = 129)			
No	11	79	68 (53%)
Some	33	46	
Much	33	14	
Very much	63	1	
Nausea (n = 91)			
No	49	101	52 (57%)
Some	31	27	
Much	38	9	
Very much	22	3	
Response at 2 h (n = 140)		n (%)	
<50%		26 (19)	
>50%		37 (26)	
>75%		32 (23)	
100% (pain freedom)		45 (32)	
Returning to everyday activities at 2 h (n = 140)			
Yes		83 (59)	
Somewhat		30 (21)	
No		27 (20)	
Overall satisfaction at 2 h (n = 140)			
Not at all		13 (9)	
Some		17 (12)	
Much		31 (22)	
Very much		79 (57)	

## Data Availability

The data that support the findings of this study are available from the corresponding author upon reasonable request due to privacy reasons.

## Supplementary Materials

The following supporting information can be downloaded at: https://www.mdpi.com/article/10.3390/brainsci14121169/s1.
