# Supplementary material for: Real-World Open-Label Experience with Rimegepant for the Acute Treatment of Migraine Attacks: A Multicenter Pilot Study
Source: Brain Sci. 2024 Nov 22;14(12):1169. doi: 10.3390/brainsci14121169 (PMC11674050; doi:10.3390/brainsci14121169)
Supplement: Supplementary file 1 [file brainsci-14-01169-s001.zip › brainsci-3313488-supplementary.pdf]

PATIENT CODE:______

CASE REPORT FROM: RESPONSE TO VYDURA™

GENDER: F_______ M_____ **AGE**:______ years

WEIGHT:_________ **HEIGHT:**_______ YEARS WITH A MIGRAINE DIAGNOSIS:_______

HAVE YOU TAKEN IN THE PAST OF OF THE BELOW TRIPTANS AND IF YES WAS IT HELPFUL WITH YOUR PAIN?:

MAXALT- RIZATRIPTAN**:** **NO - YES** (HELPFUL): YES - NO

IMIGRAN- SUTRIPTAN: **NO - YES** (HELPFUL): YES **-** NO (ADVERSE EVENT TO ANY?): YES - NO

MIGRALIN – PITUNAL: **NO - YES** (HELPFUL): YES - NO IF YES - DESCRIBE WHICH AND HOW INTENSE

RELPAX: **NO -YES** (HELPFUL): YES - NO

BEFORE YOU TOOK **VYDURA :**

PLEASE RATE HOW MUCH INTENSE WAS YOUR PAIN **BEFORE** VYDURA (FROM 0 TO 10):

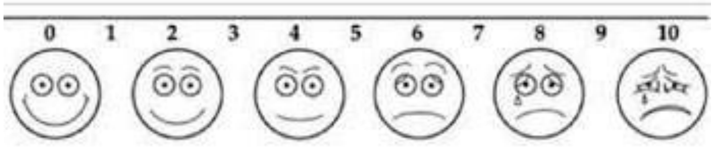

AVERSION TO LIGHT BEFORE VYDURA:	NO	LITTLE	MUCH	VERY MUCH
AVERSION TO NOISE BEFORE VYDURA:	NO	LITTLE	MUCH	VERY MUCH
NAUSEA/VOMITING	NO	LITTLE	MUCH	VERY MUCH

2 HOURS AFTER YOU TOOK VYDURA**:**

PLEASE RATE HOW MUCH INTENSE WAS YOUR PAIN AT 2H AFTER VYDURA (FROM 0 TO 10):

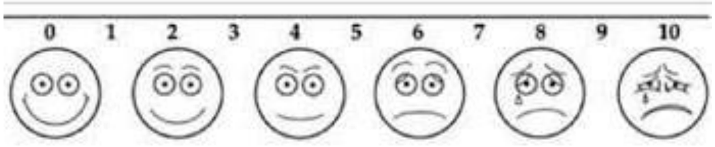

AVERSION TO LIGHT AFTER VYDURA:	NO	LITTLE	MUCH	VERY MUCH
AVERSION TO NOISE AFTER VYDURA:	NO	LITTLE	MUCH	VERY MUCH
NAUSEA/VOMITING AFTER VYDURA:	NO	LITTLE	MUCH	VERY MUCH

ADVERSE EVENTS: NO YES (DESCRIBE WHICH AND HOW INTENSE:_____________________)

- WERE YOPU ABLE TO RETURN TO YOUR DAILY ACTIVITIES/WORK AT 2HOURS POST VYDURA?:

NO SOMEWHAT - NOT QUITE GOOD YES

- PLEASE RATE YOUR OVERALL SATISFACTION TO VYDURA:

NOT AT ALL SOME MUCH VERY MUCH
